# *Mutation Reporter Tool*: An online tool to interrogate loci of interest, with its utility demonstrated using hepatitis B virus

**DOI:** 10.1186/1743-422X-10-62

**Published:** 2013-02-23

**Authors:** Trevor G Bell, Anna Kramvis

**Affiliations:** 1 Hepatitis B Virus Diversity Research Programme, School of Clinical Medicine, Faculty of Health Sciences, University of the Witwatersrand, 7 York Road, Parktown, Johannesburg, 2193, South Africa

**Keywords:** Hepatitis B virus, Mutations, Sequence analysis

## Abstract

**Background:**

An online tool, which extracts and summarises nucleotide or amino acid sequence data at specified loci of interest, was developed and tested using the basic core promoter/precore (BCP/PC) region of the hepatitis B virus (HBV). The tool is aimed at researchers without specialist computer skills.

**Methods:**

The tool consists of a web-based front-end, with a CGI script, which runs Python code to generate an output web-page. The Python code searches the input sequence data for a specified anchor motif, after which it generates summary tables and graphs of residue and motif distributions.

**Results:**

After the user provides an input file in FASTA format containing aligned sequence data (nucleotides or amino acids) and specifies an anchor motif at a known coordinate, the tool summarizes the nucleotides or amino acids at the specified loci, their frequency and analyzes motif patterns of the loci.The tool can output a graph that displays the frequency of mutations relative to a reference sequence. The tool was used to analyze the BCP/PC region of HBV belonging to subgenotypes A1, A2 and subgenotype D and to serotype HBV. The “Discovery Mode” ignores conserved loci and assists in identifying potential loci of interest.

**Conclusions:**

Although HBV was used to demonstrate the utility of the *Mutation Reporter Tool*, the tool has wide application as it is genome-agnostic: nucleotide or amino acid sequence data from any organism can be processed. Rapid characterisation of many sequences can be achieved easily when the loci of interest are known. The tool is available online, without charge, at http://hvdr.bioinf.wits.ac.za/tools

## Background

### Example organism: Hepatitis B virus

Hepatitis B virus (HBV) is one of the most important blood-borne pathogens and is endemic to the sub-Saharan African and southeast Asian regions. Worldwide, around 2 billion people have been exposed to the virus, 240 million are chronically infected, and more than half a million die annually from infection-related liver diseases [1]. At approximately 3,200 nucleotides, the HBV genome is small and has been well-characterized. The genome codes for seven different proteins from four overlapping reading frames (ORFs). To date, nine different genotypes of HBV have been identified: A to D [2,3], E and F [3-5], G [6], H [7], I [8-12] and genotype J has recently been proposed [13]. Subgenotypes have been recogonized in genotypes A to D, F and I, and these are named numerically [14]. Disease progression, clinical manifestation of illness and treatment response differ between these genotypes [15-17].

Mutations (single nucleotide polymorphisms, or “SNPs”) in the genetic sequence of HBV are common, as the virus polymerase lacks proof-reading ability [18]. Patterns of mutations at various known loci have been used to characterize the virus [14]. Certain patterns are characterstic of a particular genotype, or subgenotype [14,19], and can therefore be used to identify, or “genotype”, a given sample. Patterns at other loci are characteristic of known drug-resistant mutants [20], or indicate other important characteristics, such as down-regulation of, for example, hepatitis B e antigen (HBeAg) [21]. Therefore, the examination of nucleotides at one or more known loci, either together or individually, is routinely used to characterise HBV sequences. Identification of mutations of interest is not always straighforward, however, for a number of reasons. Firstly, the HBV genome is circular (numbered from position “1” at the Eco*R*1 restriction site), but sequence data is linear, and position “1” lies within a region of interest, which is typically sequenced both downstream and upstream of this position. Secondly, HBV genotypes are not the same length, ranging from 3182, for genotype D, to 3248 nucleotides for genotype G. Thirdly, insertions and/or deletions of varying length may be present in some isolates, or, fourthly, isolates may be recombinants of two or more known genotypes. Thus, automated analysis of the genome is complex and sequence data should be carefully curated.

#### Basic core promoter/precore (BCP/PC) mutations

HBeAg is a non-particulate secretory protein expressed by HBV. The pre-core/core open reading frame encodes for HBeAg [22]. The basic core promoter (BCP), which covers the distal X region and the proximal pre-core (PC) region, directs transcription of PC mRNA, which is translated into the pre-core/core fusion protein that is the precursor of HBeAg. This protein has a signal peptide at its amino end that targets it to the endoplasmic reticulum, where it is post-translationally modified by truncation at a fixed site on its amino end and at variable sites on its carboxyl end [21]. Various mutations within the BCP and PC regions affect the expression of HBeAg at the transcriptional, translational and post-translational levels [23,24]. The BCP A1762T/G1764A mutations affect transcription of the PC mRNA [25]. Mutations that affect HBeAg expression at the translational level include Kozak sequence (1809-1812) mutations and the G1896A stop codon mutation. Substitutions at 1809-1812 are found mainly in subgenotype A1. HBeAg expression is impaired by Kozak mutations by a leaky scanning mechanism [26]. The classical G1896A transition leads to a tryptophan to stop codon mutation, which results in the truncation of HBeAg precursor and abrogation of HBeAg expression [27]. The emergence of G1896A leads to the stabilization of the encapsidation signal (*ε*) on the pregenomic RNA in genotypes with 1858T, but is rarely found in strains which have 1858C [28]. At the post-translational level, the G1862T mutation, characteteristic of subgenotype A1, introduces a phenylalanine, which interferes with signal peptide cleavage and maturation of HBeAg [29]. Clinically, HBeAg is used as an index of viral replication, infectivity, severity of disease and response to antiviral treatment. Mutations that affect HBeAg expression are clinically relevant [17] and thus analysis of their distribution is important. We demonstrate the utility of the *Mutation Reporter Tool* using the BCP/PC mutations as an example.

### Loci of interest and patterns of residues

Analysis of loci of interest, which may be dispersed across the genome, and the resulting patterns of these loci, has traditionally been a manual, interactive process, which is time-consuming and error-prone. A new online tool, the *Mutation Reporter Tool*, has been developed to rapidly and easily display loci of interest and patterns of residues for any sequence data (nucleotides or amino acids) submitted by the user. Feedback from members of the *Hepatitis Virus Diversity Research Programme*, who used development versions of the tool extensively to analyze HBV sequences, was incorporated into the present version.

## Results and Discussion

The *Mutation Reporter Tool* is one component of a larger project currently in progress, and makes use of a common (shared) Python computer language module, consisting of a “Sequence” class, which contains several methods. The tool consists of a web-based front-end, with which the user interacts, and a CGI script, which runs the Python code and generates the output web-page. The tool has been developed to assist scientists with data analysis and does not require any specialist computer skills or installation. A detailed online tutorial is available. HBV sequence data will be used to demonstrate the utility of the tool.

### Usage

**Figure 1:**
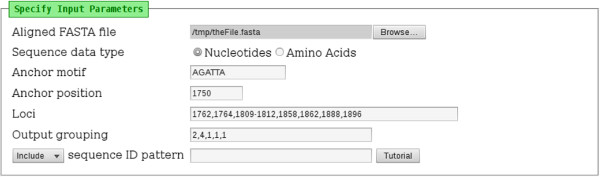
**The input interface of the Mutation Reporter Tool.** A FASTA input file of sequence data is specified. These sequence data (nucleotides or amino acids) may be genomic or subgenomic fragments. An “anchor motif”, which is common to all sequences, is provided. The “anchor position” specifies the genomic co-ordinate of the start of the “anchor motif”, such that the downstream loci of interest can be specified accordingly. In this figure, nine loci of interest have been specified. These nine loci will be grouped into columns according to the “output grouping” field: a column containing the first two loci, followed by a column containing the next four loci, followed by a column containing the final locus. Specific sequences in the input file can be included or excluded by entering a regular expression into the appropriate field. This field is blank in the figure, which indicates that all sequences will be included.

A section of the input interface of the tool is shown in Figure 1. An input file in FASTA format containing aligned sequence data (nucleotides or amino acids) is specified. The loci of interest are specified relative to a known “anchor motif” at a known genomic co-ordinate. The location of the first occurrence of the whole anchor motif in the first sequence in the file is used as the position from which the *specified* loci are determined. The loci of interest are specified as comma-separated integers without spaces (for example: 1762,1764) and/or dash-separated ranges of integers without spaces (for example: 1809-1812).

For example, the basic core promoter/precore (BCP/PC) region of HBV is routinely sequenced. Within this sequence fragment of approximately 500 nucleotides, the highly-conserved motif “AGATTA” is found at co-ordinate 1750. A file containing aligned BCP/PC sequence data is submitted to the tool with “AGATTA” specified as the anchor motif and “1750” as the anchor position. Loci of interest *downsteam* of 1750 are then specified by their absolute (and known) co-ordinates in the genome. Loci, which are known to affect the expression of HBeAg, are found at 1762, 1764 and 1896. In subgenotype A1, the “Kozak” sequence, which modulates the translation of HBeAg, is located at position “1809-1812”. All these loci are therefore entered into the “Loci” field as “1762,1764,1809-1812,1896”. Only these loci are extracted from each sequence in the input file and included on the output page.

The loci of interest can optionally be grouped into columns according to the “Output grouping” field. The field accepts a comma-separated list of integers, which indicate the number of loci to group into one output column. If no output grouping is specified, the tool will output all loci into one output column. Using the previous example of loci and an output grouping of “2,4,1”, the output would place the nucleotides at 1762 and 1764 together into one column (specified by the output grouping of “2”), the Kozak sequence at 1809-1812 into another column, and nucleotide at 1896 into a third column.

If only some sequences from the input file are to be processed, a “regular expression” can be entered next to the “Include/Exclude” drop-down box. This will then either include (or exclude) sequences for which the FASTA ID matches (or does not match) the regular expression provided. A tutorial describing regular expressions is linked from the input page for reference. Subsets of sequence data stored in one FASTA file can therefore easily be analysed separately, without having to create additional files. FASTA IDs in the output are truncated to the number of characters specified on the input page. If “Output percentages” is not selected, absolute counts are given as output, instead of percentages.

#### Output

**Figure 2:**
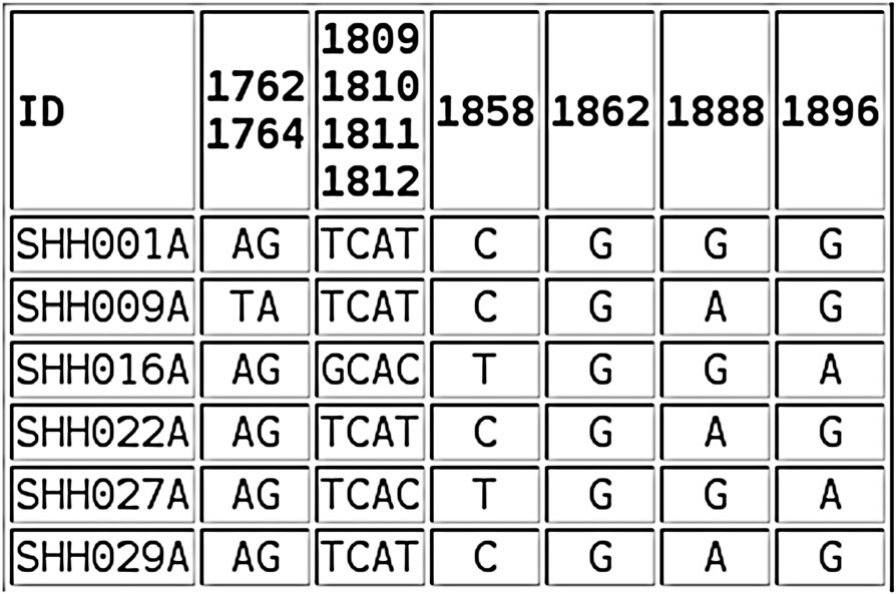
**Loci distribution.** The nucleotides at each of the specified loci within the BCP/PC region for each sequence in the input file are shown in this table. The loci are grouped into columns as specified.

**Figure 3:**
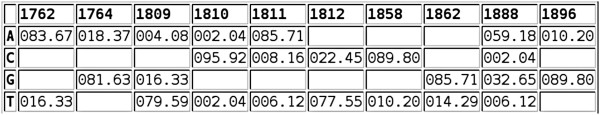
**Residue distribution summary.** The distribution of residues at each of the loci is shown, either as a percentage or as a raw count, depending on the parameter specified on the input interface (percentages are shown in this figure). Ambiguous (degenerate) bases and gaps, if present in the sequence, would also be included in the table.

**Figure 4:**
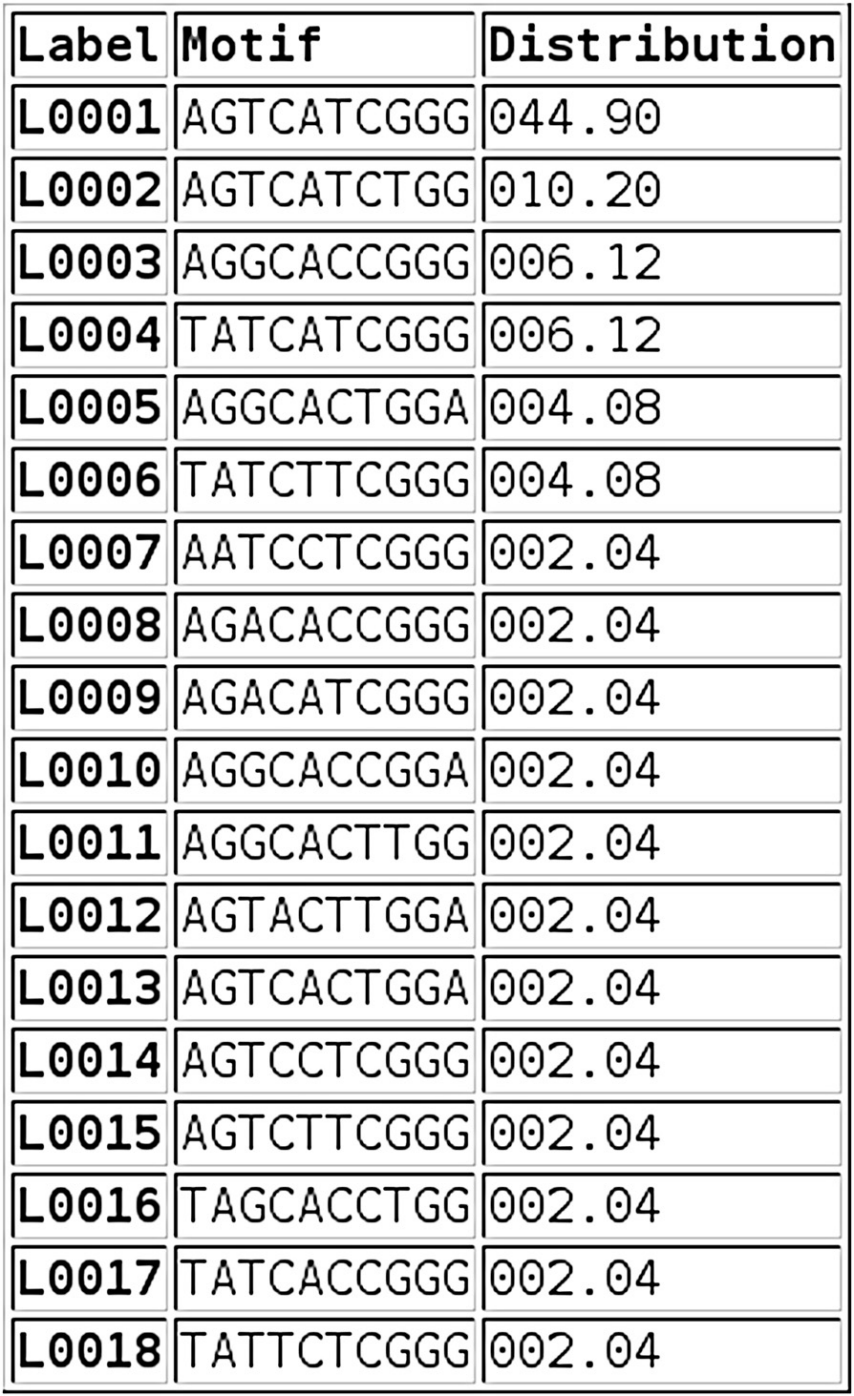
**Motif distribution.** This table shows the distribution, as a percentage, of each unique motif created by placing all the specified loci next to each other in order.

The tool produces several tables of output. The first (Figure 2) shows the residue at each of the specified loci for each sequence in the input file. The loci are grouped into columns as specified by the output grouping. The next output table shows the distribution of each residue at each locus (as a raw count or a percentage, Figure 3). Figure 4 shows part of the next table of output, which reports the number of occurrences (as a percentage, sorted in ascending order) of each unique motif pattern, as created by placing all of the specified loci next to each other in the order specified. This motif pattern can be used to classify sequences into groups and to identify the motif, which occurs most frequently. A graph of the motif distribution is displayed below the table. The raw data used to create this graph can be downloaded as a CSV file. A link below the final output table (Figure 4) opens a new page, which shows the FASTA ID associated with each of the motif patterns. This output is grouped by motif pattern for reference.

#### Example Usage: HBV serotypes

In addition to genotypic classification, HBV strains can be classified into one of nine serological subtypes (serotypes) [30]. This classification is determined by the amino acids present at either three or five known positions within the HBV surface antigen (HBsAg) [31-34]. HBV serotype is loosely correlated with genotype [19]. A published decision tree summarizes the interpretation of the amino acid positions to detemine the HBV serotype [34]. Translated (amino acid) sequence data covering the HBV surface gene can be submitted to the *Mutation Reporter Tool* with the five amino acid positions of interest (122, 160, 127, 159 and 140) specified. An output grouping of “1,1,1,1,1” should be specified to place each amino acid into its own column for easier reading. The amino acids at each position for each sequence can then be examined together with the decision tree to determine the HBV serotype.

### Mutation distribution graph

A “Reference motif” can be specified on the input page. This motif should include the reference (“wild-type”) residue for each of the specified loci, in order. For example, if loci “1809-1812,1896” are specified and the input file consists of HBV subgenotype A1 sequences, the reference motif would be “TCATG”. If a reference motif is specified, the output page will include a graph, which indicates the percentage of non-reference (mutant) residues present at each locus. If the input sequence does not contain the ambiguous base “N”, then specifying a reference motif consisting only of “N” characters will result in the tool including all of the residues at each locus, as all residues will not match the reference residue of “N” at each locus. Additional parameters on the input page are used to customize the graph appearance. These include specifying the graph dimensions (in pixels). Loci at which all sequences contain only the reference residue can be suppressed by selecting the appropriate control on the input page. Selecting the “Y-Axis scaled to 100%” control will ensure that the Y-axis of the graph extends from 0% to 100%. This is useful when preparing several graphs which are to be compared with each other. If this control is not selected, the Y-axis will be scaled according to the input data. The raw data used to construct the graph can be downloaded in CSV format from a link on the output page.

#### Example analysis: Subgenotypes A1 and A2, and genotype D

**Figure 5:**
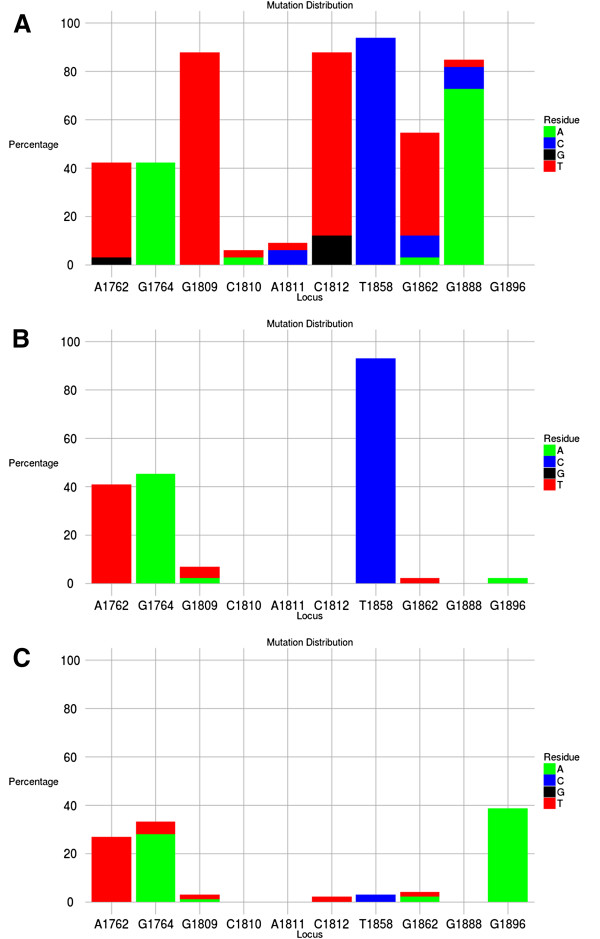
**Mutation distribution graph.** Mutation distribution graphs showing the percentage of mutant residues relative to the reference motif found at ten loci of interest specified (1762, 1764, 1809-1812, 1858, 1862, 1888, 1896). Three data sets were submitted to the tool to produce the three graphs. Panel **A** shows the mutation distribution for 33 subgenotype A1 samples, panel **B** for 34 subgenotype A2 samples and panel **C** for 93 genotype D samples. The reference motif used was AGGCACTGGG. This is also shown by the letter preceding each locus on the X-axis. To facilitate direct comparisons between the graphs, conserved loci were not suppressd and the Y-axis was scaled to 100% by selecting the appropriate controls on the input page.

A comparison of the BCP/PC region of subgenotypes A1, A2 and genotype D is depicted in Figure 5. The nucleotide at position 1858 can differentiate between genotypes A and D. Genotype A has 1858C (Figure 5A and B), whereas genotype D has 1858T (Figure 5C). The presence of 1858C precludes the development of the G1896A mutation because this would destabilize *ε* and compromise the replication of the virus [28]. On the other hand, in genotype D, with 1858T, the G1896A mutation would stabilize *ε* because of the formation of a Watson-Crick base pair between 1858T and 1896A. Thus, as is demonstrated in Figure 5, the G1896A mutation is found in genotype D but not in genotype A. The G1896A leads to a tryptophan to stop codon mutation, which results in the truncation of HBeAg precursor and abrogation of HBeAg expression [27]. G1762T/1764A mutations, which affect the expression of precore mRNA and cause a reduction in HBeAg expression can develop in genotypes A and D. However, because G1896A rarely occurs in genotype A, the frequency of 1762T/1764A is higher in this genotype compared to genotype D (Figure 5). This is the only mutation that can affect HBeAg expression in subgenotype A2 (Figure 5B), whereas in subgenotype A1 there are additional mutations that can modulate HBeAg expression (Figure 5A). In subgenotype A1, which is also characterized by 1888A, TCAT instead of GCAC occurs at position 1809-1812 (Figure 5A). This change in the Kozak sequence, preceding the precore start codon at 1814, impairs HBeAg expression by a leaky scanning mechanism. The effect of the Kozak mutations on HBeAg expression is comparable with that of the A1762T/G1764A. Co-existence of 1762T1764A and Kozak mutations reduces HBeAg expression in an additive manner [26]. Thus it can be seen that subgenotype A1 has alternative mechanisms for reducing HBeAg expression compared to genotype D, and subgenotype A2 does not develop mutations that can abrogate HBeAg expression. These differences correlate with the findings of an earlier study, which showed that the prevalence of HBeAg in serum was significantly lower in carriers of subgenotype A1 than in carriers of A2 or D [35].

### Discovery mode

When the “Discovery Mode” option on the input page is selected, the tool examines the distribution of residues at each of the specified loci and selects for processing only those loci which are *not* conserved across all input sequences. This mode can be used to “discover” loci of interest by specifying a range of loci, such as “1-100” for example, rather than specific, known loci. The tool will then examine the residues at loci 1 to 100, and will include for further processing and output only those loci at which two or more (different) residues are found. Loci at which only one residue is found will be excluded from the analysis entirely.

When “Discovery Mode” is selected, the “Output grouping”, “Reference motif” and graphing parameters are disabled, as the number and position of loci, which would be included in the final analysis is only known after the tool has processed the file. Also, as this is a “discovery mode”, it will not be known in advance which loci should logically be grouped together as a unit of interest.

### Limitations

A limitation, by design, is that the sequence data must be aligned. The position of the anchor motif in the first sequence is taken as the anchor position, and loci in all sequences are referenced according to this position. If the input sequence data are not aligned, or if the anchor motif is incorrect, the tool may return incorrect data. Whilst the number of loci, which can be specified, is not limited, it may not be feasible to enter more than a few dozen loci, as this generates a large amount of output data. All loci values must be greater than the anchor position. Updates to the tool will be made to address limitations as necessary.

## Conclusions

As an online tool, available free of charge, no download or installation is required. As demonstrated, this tool can be used for both genotyping and serotyping of HBV without the requirement of computer skills or knowledge of phylogenetics. However, as the tool is genome-agnostic, it has a wide application and nucleotide or amino acid sequence data from any organism can be analysed. Loci of interest, which may be located many hundreds of residues apart, can easily be extracted and their distribution summarised. Rapid characterisation of many sequences, or subsets of sequences, can be achieved easily when the loci of interest are known. Using the “Discovery Mode”, conserved and therefore uninformative loci, are automatically ignored, and potential loci of interest can be found and identified.

## Methods

The *Mutation Reporter Tool* consists of a web-based front-end (“client” interface) with which the user interacts, and a CGI (common gateway interface) script on a server, which runs Python language [36] code to generate the output web-page. The tool is one component of a larger project currently under development, which makes use of a common, shared Python library. The input FASTA file which the user specifies is saved locally (on the server) by the CGI script and then processed by the Python library. Methods within this library are responsible for loading sequence data from a FASTA file, processing the input parameters, and extracting the requested data from the FASTA file. The output HTML page is written to disk by the Python script. The optional output graphs are generated using the *ggplot2* graphics library [37] in the R statistical programming language [38]. If graphs are requested by the user, the Python script writes the relevant data to disk as a CSV (comma-separated value) file. A short R script, which is customized based on the input parameters specified, is also written to disk. The Python script then calls the R script, which generates the graph and writes it to disk. The images are then linked on the output HTML page. The tool is an online resource, which requires a client browser to connect to the tool’s web-server. As such, there is no stand-alone, offline version available for download.

The tool, which assumes that the submitted sequence data is aligned, finds the first occurrence of the anchor motif in the first sequence in the input file. The first character of the anchor motif is then considered to be at the position specified as the anchor position. Sequence data at each of the specified loci for all sequences in the file is then accessed and tabulated. Loci positions are mapped to positions in the sequence data using the anchor motif as an offset value. Data from the loci specified are grouped into columns according to the “output grouping” field. If this field is not specified, all loci are grouped into one output column. If a sequence ID pattern was specified, the tool executes the appropriate regular expression match on the FASTA IDs in the input file. In “Discovery Mode”, loci at which no variation is found are excluded.

## Competing interests

The authors declare that they have no competing interests.

## Authors’ contributions

AK is the principle investigator. TB conceived the idea of the tool, wrote the code, established and maintained the server, software and hardware. TB and AK wrote the paper. Both authors read and approved the final manuscript.
